# The effect of biomechanical foot-based interventions on patellofemoral joint loads during gait in adults with and without patellofemoral pain or osteoarthritis: a systematic review protocol

**DOI:** 10.1186/s13047-022-00596-7

**Published:** 2022-12-13

**Authors:** Samual A. Kayll, Rana S. Hinman, Kim L. Bennell, Adam L. Bryant, Patrick L. Rowe, Kade L. Paterson

**Affiliations:** Centre for Health, Exercise and Sports Medicine, Department of Physiotherapy, School of Health Sciences, The University of Melbourne, 3010, Level 7, Alan Gilbert Building, 161 Barry Street, Parkville, Australia

**Keywords:** Foot-orthoses, Footwear, Taping, Patellofemoral joint, Stress, Pressure, Moment, Force, Biomechanics

## Abstract

**Background:**

Patellofemoral pain is highly prevalent across the lifespan, and a significant proportion of people report unfavourable outcomes years after diagnosis. Previous research has implicated patellofemoral joint loading during gait in patellofemoral pain and its sequelae, patellofemoral osteoarthritis. Biomechanical foot-based interventions (e.g., footwear, insoles, orthotics, taping or bracing) can alter patellofemoral joint loads by reducing motions at the foot that increase compression between the patella and underlying femur via coupling mechanisms, making them a promising treatment option. This systematic review will summarise the evidence about the effect of biomechanical foot-based interventions on patellofemoral joint loads during gait in adults with and without patellofemoral pain and osteoarthritis.

**Methods:**

MEDLINE (Ovid), the Cumulative Index to Nursing and Allied Health Literature CINAHL, The Cochrane Central Register of Controlled Trials (CENTRAL), SPORTdiscus (EBSCO) and Embase (Ovid) will be searched. Our search strategy will include terms related to ‘patellofemoral joint’, ‘loads’ and ‘biomechanical foot-based interventions’. We will include studies published in the English language that assess the effect of biomechanical foot-based interventions on patellofemoral joint loads, quantified by patellofemoral joint pressure, patellofemoral joint reaction force and/or knee flexion moment. Two reviewers will independently screen titles and abstracts, complete full-text reviews, and extract data from included studies. Two reviewers will assess study quality using the Revised Cochrane Risk of Bias (RoB 2) tool or the Cochrane Risk Of Bias In Non-Randomized Studies – of Interventions (ROBINS-I) tool. We will provide a synthesis of the included studies’ characteristics and results. If three or more studies are sufficiently similar in population and intervention, we will pool the data to conduct a meta-analysis and report findings as standardised mean differences with 95% confidence intervals. If a meta-analysis cannot be performed, we will conduct a narrative synthesis of the results and produce forest plots for individual studies.

**Discussion:**

This protocol outlines the methods of a systematic review that will determine the effect of biomechanical foot-based interventions on patellofemoral joint loads. Our findings will inform clinical practice by identifying biomechanical foot-based interventions that reduce or increase patellofemoral joint loads, which may aid the treatment of adults with patellofemoral pain and osteoarthritis.

**Trial registration:**

Registered with PROSPERO on the 4th of May 2022 (CRD42022315207).

**Supplementary Information:**

The online version contains supplementary material available at 10.1186/s13047-022-00596-7.

## Background

Patellofemoral pain is highly prevalent from adolescence to adulthood, regardless of activity level, health status or demographic [1]. Unresolved patellofemoral pain is insidious due to the accompanying psychological distress, reduced quality of life [2] and increased likelihood of patellofemoral osteoarthritis [3]. Clinical guidelines characterise patellofemoral pain as pre- or peri-patellar pain during activities that load the patellofemoral joint, such as squatting or descending stairs [4, 5]. Furthermore, adults with patellofemoral pain walk with increased patellofemoral joint loads [6, 7] when compared to healthy controls. This is important given increased loading during gait may contribute to the structural progression of patellofemoral osteoarthritis [8]. Accordingly, identifying interventions that can reduce patellofemoral joint loads during gait may be a promising treatment option for patellofemoral pain and osteoarthritis.

Clinical practice guidelines recommend education on load management and kinesiophobia, and exercise targeting the thigh and hip musculature to treat patellofemoral pain [4, 9]. However, 57% of people still report symptoms 5-8 years following diagnosis, possibly due to the low adherence rate with exercise (20% in one randomised controlled trial [10]). As an alternative (or adjunct), clinicians will commonly utilise biomechanical foot-based interventions (e.g., footwear, insole, orthotic, tape or brace placed on the foot). In addition to being low burden and widely available, these interventions can alter patellofemoral joint loads via joint coupling mechanisms. For example, footwear with a lower pitch (i.e., difference in height between the heel and forefoot of the shoe) reduces step length, bringing the stance limb closer to the centre of mass, reducing knee flexion and the quadricep moment arm, thereby reducing patellofemoral joint load [11, 12]. Additionally, foot orthoses, taping and bracing can reduce rearfoot pronation [13, 14], a motion theoretically linked to an increase in patellofemoral joint load via increased femoral internal rotation and subsequent compression between the lateral trochlea and patella [15].

Previous studies quantify patellofemoral joint loads using pressure [12, 16, 17], reaction force [18, 19], or surrogate measures such as the external knee flexion moment [20–22]. The patellofemoral joint reaction force is the resultant compressive force from the pull of the patella tendon and the quadriceps [23]. The degree of reaction force is dependent on knee flexion, whereby greater knee flexion leads to a greater reaction force [24]. Patellofemoral joint pressure is the reaction force divided by the unit of contact area between the patella and trochlea groove. While the reaction force increases with knee flexion (due to the greater compressive force from the patella tendon and quadriceps), so does the contact area [25]. The increase in contact area helps to offset the increasing reaction force during tasks with high knee flexion (e.g., a deep squat), allowing the patellofemoral joint to accommodate these forces safely. However, the increase in contact area does not totally offset the increase in reaction force, resulting in a net gain in patellofemoral joint pressure [24], highlighting the intricate relationship between patellofemoral joint reaction force, contact area and pressure. Accordingly, it is essential to consider both patellofemoral reaction force and pressure to understand the effects of interventions designed to reduce patellofemoral joint loads.

In vivo measurement of patellofemoral joint pressure and reaction force currently requires impractical instrumented knee joint implants [26]. As such, to estimate reaction force, studies typically use kinematic and kinetic data to inform a 2D or 3D model of the knee that contains representations of the quadriceps lever arm, quadriceps muscle force and the relationship between the quadriceps muscle force and patellofemoral joint reaction force [27]. To additionally estimate patellofemoral joint pressure, studies have estimated contact area based on data obtained from cadavers, healthy people, or people with patellofemoral joint pain [27].

To date, there has been no systematic review specifically evaluating the effects of biomechanical foot-based interventions on patellofemoral joint loads. Although one systematic review [18] included some studies that evaluated foot-based interventions, its focus was on comparing patellofemoral joint reaction force (only) across everyday activities and it did not conduct a sub-group analysis of intervention effects. As a result, the specific effect(s) of biomechanical foot-based interventions on patellofemoral joint loads remains unclear.

## Objective

The objective of this study will be to systematically review the literature to evaluate the effect of biomechanical foot-based interventions on patellofemoral loads (as measured by pressure, reaction force and external knee flexion moment) during gait in adults with and without patellofemoral pain and osteoarthritis.

## Methods/design

Our protocol is guided by the Methodological Expectations of Cochrane Intervention Reviews (MECIR) standards [28] and Preferred Reporting Items for Systematic Review and Meta-Analysis Protocols (PRISMA-P) 2015 checklist [29, 30]. For the PRISMA-P checklist, see Additional file 1. The systematic review protocol was prospectively registered with the International Prospective Register of Systematic Reviews (PROSPERO) on the 4th of May 2022 (CRD42022315207). We will report any changes to our protocol when we publish our review findings.

### Inclusion criteria

#### Types of studies

We will consider published original research for inclusion. Only studies published in the English language will be considered due to the lack of language translation resources. Study designs may include, but are not limited to, case-control, cross-sectional, cross-over, or randomised controlled trials that assess the effects of biomechanical foot-based interventions on patellofemoral joint loads in humans. Editorials, comments, letters, abstracts, review articles, theses, and dissertations will be excluded. Study selection criteria were established *a priori* using the Population, Intervention, Comparison, Outcome (PICO) framework [31]. Studies that fulfil the following criteria will be included.

Population: We will include studies of adults aged 18 years or older of any sex. Participants in eligible studies may be healthy (free from any condition that may affect gait) or have a diagnosis of patellofemoral pain or patellofemoral osteoarthritis. We will accept any study whereby the inclusion criteria defined participants as having patellofemoral pain or patellofemoral osteoarthritis. No restriction will be placed on the severity of either condition. Studies will be ineligible if participants have a predominant lower limb condition or surgery that affects gait (e.g., anterior cruciate ligament rupture/repair, tibiofemoral osteoarthritis, hip osteoarthritis, or joint arthroplasty) or predominant comorbidities (e.g., stroke or Parkinson’s). Where mixed populations of participants are reported (e.g., people with tibiofemoral osteoarthritis and patellofemoral osteoarthritis), only studies with 80% or more participants that meet the above criteria will be included.

Intervention: Following the Template for Intervention Description and Replication (TIDieR) checklist [32], we will consider any biomechanical foot-based intervention that has the objective of reducing patellofemoral joint loads during gait (e.g., walking or running) in a biomechanical laboratory. We define a biomechanical foot-based intervention as any type of footwear, insertable shoe worn device (i.e., orthotic or insert), ankle brace, wedge or foot/ankle taping that is designed to alter patellofemoral joint loads. Biomechanical foot-based interventions of any duration will be eligible for inclusion (e.g., studies evaluating immediate effects and studies with longer-term durations). Other interventions such as surgery, exercise, or manual therapy will be excluded, including when combined with a foot-based intervention, such as exercise and foot orthoses.

Comparator: Studies will be eligible for inclusion if they have compared an eligible biomechanical foot-based intervention to either: (1) no intervention (i.e., no biomechanical foot-based intervention) and/or (2) any other eligible biomechanical foot-based intervention. Studies that only use a barefoot comparator will be excluded.

Outcome: Studies will be eligible for inclusion if they have measured patellofemoral load via 2D or 3D motion analysis during gait. Patellofemoral joint loads must be quantified using either: (1) patellofemoral joint pressure (patellofemoral joint reaction force divided by a unit of contact area), (2) patellofemoral joint reaction force (resultant compressive force from the pull of the quadriceps and patella tendon), or (3) external knee flexion moment (or its equivalent internal knee extension moment) during stance (combination of the ground reaction force and the perpendicular distance of this force from the joint centre). There are numerous ways to report pressure, force, and moment thus we will accept any units of measurement. Any other kinetic or kinematic outcome will be excluded (e.g., We have chosen these parameters as it is known that people with patellofemoral pain reduce their knee flexion moment, force and therefore pressure, possibly to reduce pain [33].

### Methods for identification of studies

Following the Methodological Expectations of Cochrane Intervention Reviews (MECIR) standards [28], we will conduct a comprehensive literature search that includes five electronic databases, including MEDLINE (Ovid), the Cumulative Index to Nursing and Allied Health Literature (CINAHL), The Cochrane Central Register of Controlled Trials (CENTRAL), SPORTdiscus (EBSCO) and Embase (Ovid). These databases were selected to ensure a comprehensive retrieval of relevant studies, and because they are recommended for health topic searches [28]. We will use free text and indexed terms for the population, intervention and outcome components as defined above. The search strategy for MEDLINE (Ovid) is presented in Appendix 1, and we will adapt this strategy for the remaining databases with the help of an academic librarian from The University of Melbourne. The search terms used are based on other similar reviews [18, 28, 34]. We will not apply any limits on the type or publication date of the studies. In addition, we will manually check the reference lists of all included studies and relevant systematic reviews to identify any additional potentially eligible studies that may have been missed. We will follow the recommendation of Adams et al. [35] to exclude grey literature when an academic field is relatively mature and not systematically search any grey literature.

### Data collection and analysis

The search results from each database will be downloaded in a RIS file and uploaded into Covidence systematic review software (Veritas Health Innovation, Melbourne, Australia; available at www.covidence.org) for study screening and to remove duplicates. Study selection will be completed using Covidence.

#### Selection of studies

One reviewer will conduct the initial search (SAK), followed by a two-step screening process. First, two reviewers (SAK and PLR) will independently assess the titles and abstracts of all identified studies using *a priori* inclusion and exclusion criteria to determine their potential eligibility and exclude any irrelevant studies. Second, the two reviewers will independently apply *a priori* inclusion and exclusion criteria to the full texts. Studies deemed eligible by both reviewers will be included in the review. Any disagreements between reviewers at either step will be resolved through consensus with a third reviewer (KLP). When necessary, we will correspond with the authors to clarify study eligibility. We will use a PRISMA flow diagram to document our searching, screening, and selecting of studies for inclusion. Publications with similar names and dates will be compared to identify data duplication. Where it is evident that the same data are presented (e.g., same baseline data), duplicate data with the highest methodological quality index score will be included. If there is uncertainty over the duplicate data, the authors will be contacted for clarification.

#### Data extraction

Two reviewers (SAK and PLR) will independently extract relevant data. We will use a structured pre-piloted electronic data collection form. A third reviewer will resolve any discrepancies. We will extract the following descriptive data from each study:

- Study characteristics: Sample size, inclusion/exclusion criteria, and year of publication.
- Participant characteristics: Age, sex, socio-demographic and whether they were healthy or diagnosed with patellofemoral pain or osteoarthritis. If available, we will extract the severity of the disorder according to the relevant measure used (e.g., Kellgren and Lawrence grading system for osteoarthritis).
- Intervention and comparator characteristics: Information regarding the footwear, insertable shoe worn devices, taping or bracing used as the intervention and comparator, including but not limited to the author’s description, type, brand, version, heel thickness, pitch, motion control properties and sagittal rigidity and angulation. In addition, the gait speed, and the surface (e.g., treadmill or runway) used for gait analysis will also be extracted. For the insertable shoe worn devices, bracing and taping interventions, we will also extract information on the type of shoes (as above) that participants wore during gait analysis, including for the comparator condition. Consistent with a previous review [36], if a study investigates multiple variations of a given intervention, we will extract the intervention variant postulated to have the maximal biomechanical effect and the control condition postulated to have the minimal biomechanical effect. For example, where a study investigates foot orthoses with different degrees of wedging (e.g., 5 vs. 10 degrees), we will extract data relating to the 10-degree orthotic as the intervention condition. The opposite will be the case for the comparator. We will classify the footwear and insertable shoe-worn device based on the author’s description. If there is insufficient detail, we will source it from the manufacturer online.
- Patellofemoral joint load outcomes: All available data on the patellofemoral joint loads from each study’s intervention and comparator arm will be extracted. We have established a predefined decision rule in case a study reports multiple eligible outcome measures. Specifically, we will use the following hierarchy to extract data regarding the outcome that best represents patellofemoral joint loads: (1) patellofemoral joint pressure, (2) patellofemoral joint reaction force and (3) knee flexion moment. All outcomes must be the peak during stance. We will also accept studies that report early stance peak pressure, reaction force or moment, as during normal walking and running gait peak patellofemoral joint loads are found in early stance [7, 37]. Where a study reports both early stance and overall peak values, we will extract the overall peak value.

We expect that the outcomes of interest will be reported as continuous data. We will extract the point estimate and include its method of statistical analysis. Additionally, we will extract the corresponding measure of variability (standard deviation, standard error, *p*-value or 95% confidence interval). To make comparisons of individual studies, we will analyse the data based on the mean, standard deviation, and the number of people in the intervention and comparison groups to calculate the mean difference and 95% confidence intervals.

### Assessment of methodological quality of included studies

Two reviewers (SAK and PLR) will independently assess the methodological quality of the included studies using the Revised Cochrane Risk of Bias tool for randomised trials (RoB 2) [38] or the Cochrane Risk Of Bias In Non-Randomized Studies – of Interventions (ROBINS-I) tool [39] for non-randomised trials. For the RoB 2, we will consider five domains: (1) bias arising from the randomisation process, (2) bias due to deviations from intended interventions, (3) bias due to missing outcome data, (4) bias in measurement of the outcome, and (5) bias in selection of the reported result. Citing evidence from the study article, relevant papers, or the study authors, two reviewers will independently rate each domain as either low risk of bias, some concerns or high risk of bias. For the ROBINS-I, we will consider seven domains: (1) bias due to confounding, (2) bias in selection of participants into the study, (3) bias in classification of interventions, (4) bias due to deviations from intended interventions, (5) bias due to missing data, (6) bias in measurements of outcomes, and (7) bias in selection of the reported result. Two reviewers will then independently rate each domain as either low risk of bias, moderate risk of bias, serious risk of bias, critical risk of bias or no information. In the case of disagreement between the two reviewers, a third author (KLP) will appraise the study independently, and the research team will convene until a consensus is reached. The risk of bias of each study will be reported in the summary of findings table.

### Missing data

We will attempt to contact the study authors to request either missing data or data published in graphical form. If the authors cannot be contacted, do not respond, or decline to provide data, we will extract any graphical data using Web Plot Digitizer software (Ankit Rohatgi, California, USA; available at https://automeris.io/WebPlotDigitizer). We will address the impact of missing data in a sensitivity analysis. Where available, we will refer to study protocols and baseline publications to identify outcome data expected to be present at follow-up. If the data are absent, we will note reporting bias.

### Data synthesis and analysis

According to the Cochrane Handbook for Systematic Reviews of Interventions [28], if any unanticipated issues arise, we will make sensible post-hoc decisions about excluding studies. These will be documented in the review findings, possibly accompanied by a sensitivity analysis.

We will pool data across studies that are sufficiently similar in population, interventions (e.g., minimalist footwear, motion control footwear, arch support orthoses or medial wedge device) and comparator. Consistent with a previous review [36], aspects of the same outcome that are classified by different timepoints but produce the same value (e.g., overall peak patellofemoral joint pressure and peak patellofemoral joint pressure during stance) will be pooled across studies. We will pool outcomes regardless of measurement method. Where there are three or more studies that are sufficiently similar, meta-analysis will be performed. We will account for the expected heterogeneity among included studies using a random-effects meta-analysis [40] and the inverse variance method using Review Manager statistical software (RevMan, Version 5, Copenhagen: The Nordic Cochrane Centre, The Cochrane Collaboration, 2014). Where possible, outcomes will be reported as standardised mean differences with 95% confidence intervals. Standardised mean differences will be interpreted as: minimal < 0.2, small 0.2–0.49, medium 0.50–0.79 and large > 0.8.

We will assess the degree of heterogeneity by visually inspecting forest plots and examining the χ² test for heterogeneity. Heterogeneity will be quantified using the I² statistic, which describes the percentage of total variation across studies. This percentage will be interpreted considering the size and direction of effects and the strength of the evidence for heterogeneity, based on the *p*-value from the χ² test. I^2^ values of 30%, 50% and 75% will be considered moderate, substantial and considerable heterogeneity, respectively [28, 41]. Where heterogeneity is present in pooled estimate effects, we will explore the possible reasons for variability by conducting subgroup analyses. If we cannot perform a meta-analysis, we will conduct a narrative synthesis of results that considers potential clinical or methodological similarities and differences to explain the heterogeneity between the findings of different studies and examine patterns in the data.

Two reviewers (SAK and PLR) will independently use the Grading of Recommendations Assessment, Development and Evaluation (GRADE) approach, as described in the Cochrane Handbook, to assess the quality of the body of evidence. We will use the GRADEpro GDT application (http://gradepro.org) to produce a ‘summary of findings’ table to compare the intervention effect magnitudes on main outcomes.

### Assessment of non-reporting bias

If there are at least ten studies eligible for review, we will use funnel plots to examine small study effects and highlight the presence of any publication bias. We will perform a statistical test for funnel plot asymmetry and conduct further statistical tests to explain any asymmetry.

### Subgroup analyses

We plan to perform subgroup analyses on each type of biomechanical foot-based intervention (e.g., footwear, foot orthoses, taping or bracing). In addition, we will investigate the biomechanical foot-based intervention effects on healthy, patellofemoral pain and patellofemoral osteoarthritis populations by subgrouping these three populations.

## Discussion

Patellofemoral pain is prevalent in the general population [1] and can result in psychological distress [2] and precede the development of patellofemoral osteoarthritis [3]. Current treatments for patellofemoral pain are not effective for many patients, as evidenced by the high proportion that still report symptoms five to eight years after diagnosis [42]. This may be due to the low adherence rate with current interventions such as exercise. Patellofemoral joint loading has been implicated in patellofemoral pain [6] and may hasten disease progression in adults with patellofemoral osteoarthritis [8]. Thus, interventions that can reduce patellofemoral joint loads, such as biomechanical foot-based interventions, are a promising treatment option, particularly given they are more likely to have greater adherence than other common treatments such as exercise. Our systematic review will be the first to examine the effects of different biomechanical foot-based interventions on patellofemoral joint loads.

The strength of our review will be the inclusion of a range of foot-based interventions (footwear, foot orthoses, insoles, bracing and taping) and patellofemoral joint loading parameters (patellofemoral stress, pressure, reaction force, and knee flexion moment). As such, we will be able to determine the isolated effect of interventions designed to reduce patellofemoral joint loads via altering foot biomechanics. Although clinicians may use a combination of treatments in the clinical setting, it is important first to establish whether biomechanical foot-based interventions reduce patellofemoral joint loads in isolation before investigating cumulative effects with other treatments. A limitation of our review is the inclusion of only English language studies, and studies restricted to adult participants, albeit there is very limited research in adolescent populations.

Our findings will identify biomechanical foot-based interventions that reduce and increase patellofemoral joint loads. These findings will aid therapists when selecting appropriate biomechanical foot-based interventions for adults with patellofemoral pain and osteoarthritis.

### Supplementary Information

**Additional file 1.**

## Data Availability

Not applicable.

## Appendix 1

### MEDLINE search strategy

This is the template search strategy that will be adapted as needed to fit the other four databases to be searched in the final review. The exact search for each of the databases will be available at final publication.

Ovid MEDLINE(R) and Epub Ahead of Print, In-Process, In-Data-Review & Other Non-Indexed Citations and Daily < 1946 to March 04, 2022>.

**Table:**

1	(patellofemoral or patello-femoral or patella or knee).mp. [mp = title, abstract, original title, name of substance word, subject heading word, floating sub-heading word, keyword heading word, organism supplementary concept word, protocol supplementary concept word, rare disease supplementary concept word, unique identifier, synonyms]
2	(footwear or shoe or wedge or insole or orthotic or orthoses or minimalist or heel or insert or orthosis or taping).mp. [mp = title, abstract, original title, name of substance word, subject heading word, floating sub-heading word, keyword heading word, organism supplementary concept word, protocol supplementary concept word, rare disease supplementary concept word, unique identifier, synonyms]
3	exp Shoes/
4	exp orthotic devices/ or exp athletic tape/ or exp braces/ or exp foot orthoses/
5	2 or 3 or 4
6	(kinetics or load or stress or pressure or moment or torque or biomechanic or reaction force or quadriceps force).mp. [mp = title, abstract, original title, name of substance word, subject heading word, floating sub-heading word, keyword heading word, organism supplementary concept word, protocol supplementary concept word, rare disease supplementary concept word, unique identifier, synonyms]
7	exp Biomechanical Phenomena/
8	6 or 7
9	1 and 5 and 8
10	limit 9 to human

## Abbreviations

CINAHL: Cumulative Index to Nursing and Allied Health Literature
CENTRAL: Cochrane Central Register of Controlled Trials
RoB 2: Revised Cochrane Risk of Bias
ROBINS-I: Cochrane Risk Of Bias In Non-Randomized Studies – of Interventions
MECIR: Methodological Expectations of Cochrane Intervention Reviews
PRISMA-P: Preferred Reporting Items for Systematic Review and Meta-Analysis Protocols
PROSPERO: International Prospective Register of Systematic Reviews
PICO: Population, Intervention, Comparison, Outcome
TIDieR: Template for Intervention Description and Replication
